# Supplementary material for: Patterns of Intron Gain and Loss in Fungi
Source: PLoS Biol. 2004 Nov 30;2(12):e422. doi: 10.1371/journal.pbio.0020422 (PMC532390; doi:10.1371/journal.pbio.0020422)
Supplement: Table S1 — Also available at http://genes.mit.edu/NielsenEtAl/. (4.3 MB ZIP). [file pbio.0020422.st001.zip › NielsenEtAl/html/816.html]

AN0804.1.NCU02105.1.MG04650.1.FG00914.1


```
 CLUSTAL W (1.82) Multiple Sequence Alignments - Introns Inserted


Sequence 1: MG04650.1	211 aa
Sequence 2: FG00914.1	253 aa
Sequence 3: NCU02105.1	293 aa
Sequence 4: AN0804.1	255 aa
Alignment Length: 294 aa
Number Identitical Residues: 104 aa
Alignment Score (without introns) 5706


MG04650.1 	-----------------------------------~------------------MNASAK
NCU02105.1	MANSKFEYVKQFEQPDSLLPNTWIVVRLDGRGFTK2FSTKYAFEKPNDKRALDLMNAAAR
FG00914.1 	-----------------------------------~MCAKYAFEKPNDRRALDLMNTAAK
AN0804.1  	------------------------------MANSR2LSDRYAFEKPNDRRALDLMNAAAV
          	                               . :   .   : .....  : . **::* 

MG04650.1 	SVVTELPEITIAYGVSDEYS~FVFHKHCSL~FDRRSG2------------~---------
NCU02105.1	SVMSELPDITIAYGVSDEYS~FVFHKSCTL~FERRAS~KLVSTIVSTFTA~YYIHHWPTY
FG00914.1 	AVVTELPDITIAYGVSDEYS2FVFHKACTL~FERRAS2KLVSTVVSTFTA~NYVYFWSTH
AN0804.1  	EVLKDLPDLAIAYGVSDEYR~FDLTNSRRI1LSSIPP~---VSSLSGGAP1HYIYLWGNY
          	 *:.:**:::*********  * : :   : :.  .      :  :  :.        . 

MG04650.1 	-----PLSPP-LPSFDGRAVCFPSVQNLRDYMSWRQVDC~HINNLYNTTYWALIQQGNLD
NCU02105.1	FVDGPPLSPP-LPSFDGRAVCYPSVQNLRDYMSWRQVDC~HINNLYNTTFWALINQGGMD
FG00914.1 	FPD-TPLSPP-LPSFDGRAVCYPSVQNLRDYMSWRQVDC1HINNLYNTSFWSLIQVKGLD
AN0804.1  	FPD-TPLQPPHLPSFDGRAVVYPTIRNLRDYMSWRQVDC1HINNLYNTTFWMMVLKGGMS
          	  . .**.** ********* :*:::************* ********::* ::   .:.

MG04650.1 	AHTAERTLA0GTFAADKNEILFSKFGINYNNELDIYKKGSVVFRD0YILEEPQ---DGQG
NCU02105.1	GTAAELMLK~GTFSADKNEILFKKFGINYNNEPEMFKKGSVVFRN~YELVEPGTKKVSEE
FG00914.1 	NKEAEKRLA0GTYAADKNEILFSECSINYNNEPEIYKKGSVIFRD0YELVDP----DSHN
AN0804.1  	NTDAENELK0GTLSSDKNEILFKRFGINYNNEEEIYKKGSVVYRQ0YQLEERK-----PL
          	   **  *  ** ::*******.. .****** :::*****::*: * * :         

MG04650.1 	VAQKVEALVEPVVKSKTQAENEKKKRAKARVTVEHLDIIKDDFWDRRPWLLSNKPGEIPK
NCU02105.1	EAEEMSSSAVPEVKSKSQVEKDKKVRTKAKIVVEHLDIIRDEFWERRPWLLSGTPGKVPK
FG00914.1 	ITQTIDSQAEPTQQSKTQKEKDKKSRAKARVVVEHVDIIKDDFWDRRPWLLSNKPGKIPK
AN0804.1  	KAPEEEGPTVQESISKSQQEKLRKLRRKAQVIVEHVDIIKDEFWERRPWILSGKPGRLPA
          	 :   .. .     **:* *: :* * **:: ***:***:*:**:****:**..**.:* 

MG04650.1 	QP
NCU02105.1	EP
FG00914.1 	QT
AN0804.1  	EA
          	:.
```
